# Progressive Paralysis of the Insane

**Published:** 1868-07-01

**Authors:** A. W. Bosworth

**Affiliations:** Thesis for the Degree of M.D., Presented to the Faculty of the Rush Medical College, Chicago; Paris, France


### CHICAGO MEDICAL JOURNAL.

*Vol. XXV.—JULY* 1, 1868.—*No.* 13.

### PROGRESSIVE PARALYSIS OF THE INSANE.

(PARALYSIE GÉNÉRALE DES ALIÉNÉS OF THE FRENCH.)

BY A. W. BOSWORTH, M.A., PARIS, FRANCE.

Thesis for the Degree of M.D., Presented to the Faculty of the Rush Medical College, Chicago.

(*Concluded from page* 392.)

### DIAGNOSIS.

It is the imperious duty of the careful physician not to make too suddenly his conclusions. Nothing is more annoying than to learn that our judgment was erroneous. Nothing more embarrassing than to be able to early distinguish this sad, fatal disease from some other forms of mental derangement. In cases of doubt, then, let us learn not to be too hasty.

Usually the most difficulty will be presented in distinguishing general paralysis from that form of insanity known as simple mania, when, especially, assuming an ambitious character. As soon as we find a patient divulging ambitious delirium, we must be on our guard, remembering that this tendency is, in the immense majority of cases, closely connected with lesions of motility. All authors usually admit that mania of an ambitious type may exist independently of being connected with those terrible indications which truly reveal to us the fatal termination of general palsy.

Consequently, if our patient, with his agitation, irascibility, and ambitious ideas, does not present impediment of speech, or trouble of motility, we must carefully become informed concerning his antecedents and the circumstances which preceded the beginning of his disease. By thus searching, we shall find, if we have to do with a case of general palsy, that before the manifestation of ambitious delirium, memory was diminished; and, in fact, all the symptoms of intellectual weakness may be recognized. Especially should we ascertain if the patient has been subject to cerebral congestion, followed by difficulty in speaking.

M. Calmeil says: "We must not hasten to pronounce a diagnosis from a simple impediment of speech with or without delirium, because this may possibly arise from a deep inflammatory focus with or without a clot of blood in the cerebral substance." Ambitious monomania of the paralytic is easily distinguished from ordinary ambitious monomania. This latter is rare; the person affected with it, taciturn and morose at the beginning, realizes that he is ill; fears that he is going to lose his reason; and, at first, endeavors to banish the delirious ideas which he knows to be absurd. Later, mastered by the delirium, he conforms himself to his ideas; he imitates the character or person he has chosen; his actions and words seem consistent with the position he believes himself called to fulfill. If he considers himself general, or king, then does he use terms suitable for his illusory dignity.

Hallucinations, rare with the paralytic, are, on the contrary, common to him.

Nothing similar is seen with the victim of general palsy. His ideas are not only absurd, but they are very changeable. He, while king, and at the same moment Pope or President, speaking of his millions, will acknowledge his profession, however humble it may be, and declare he has only a few cents in the world.

The ordinary ambitious monomaniacs retain for years their systematically organized delirium, while the paralytic is continually, day by day, hour by hour, changing his absurd ideas. Finally, the absence or presence of trouble of motility, the whole course of the two diseases, are too well known to be here longer considered.

To distinguish general palsy from simple dementia, we must remember that this latter impaired mental condition occurs consecutive to some other cerebral affection, existing even for years, as from melancholia, or mania; and that by slow gradations the mental faculties have become confused, and at length obliterated. Although here we may find abolition, more or less apparent, of the intellectual, sensitive, and voluntary faculties, yet when we recall to mind that paralytical dementia has not been preceded by any mental affection; that in a few weeks, or a few months, it is so apparent as to be recognized by slight observation; that without delay there is the appearance of delirious ideas, either ambitious or melancholic, we hardly need to be obliged to base the diagnosis on the presence or absence of difficulty of speech, or trouble of motility.

In speaking of insanity, Dr. Aitken says that melancholia comes next in frequency to mania, and that the melancholic and stupid are most frequently cured or improved. Such being the case, it behooves the physician not to confound the appalling disease of general palsy of a melancholical form with melancholia of a stupid nature. To well found the diagnosis in some cases is often perplexing, as the delirious ideas are the same; dementia in both often very similar; and the stupor is accompanied with trouble of motility. We must carefully ascertain the antecedents; give especial attention to their being, or having been, cerebral congestions; and all the precursory symptoms of general palsy must be held in mind to contrast them with our doubtful case. We shall find, if the patient is laboring under general palsy, that his melancholic or hypochondriacal delirium has not only its characteristic absurdity, but occasionally there will be seen, if only momentarily, yet of vast importance, some ambitious ideas, as of immense wealth, or great fame, gliding into his train of lamentation, fear of death, or a curious state of imagined metamorphosis.

Dr. Aitken nowhere states that the absurd delirium of simple melancholia divulges the complication of ambitious ideas. M. Marcé says: "In simple melancholia the delirium, although monotonous, does not offer the same appearance of absurdity, and never is complicated with ambitious ideas."

In simple melancholia, when the stupor is intense, involuntary evacuations may occur at the very onset, while in general palsy they only slowly and progressively arrive. In melancholia, as soon as there is stupor, there is directly slowness of movement, and muscular weakness, which present a contrast with the energetic, yet stiff and badly co-ordinating movement of the paralytic during the first period. In every doubtful case the only duty, though sometimes annoying, is to delay the diagnosis till something may occur to throw light on the subject.

Not having time to lengthen this part of our subject, we will only mention that many of the inexperienced have often taken for an epileptic attack, apoplectiform or epileptiform congestions caused by general palsy; that cerebral softening, when situated in the central parts, or occupying both hemispheres, so closely presents symptoms allied to general palsy, that sometimes a *post mortem* has only revealed its true nature. Even in such cases, good authorities state that no ambitious ideas are ever seen.

### Treatment.

The physician, once feeling sure as to his diagnosis, has an important part to play in regard to the doubts and fears of the patient's relatives and friends. It is useless to here endeavor to indicate how he must comport himself; only we would remark that this disease opens to him a wide field to employ a large amount of good sound judgment and advice. Let us be cautious not to be led astray, nor allow friends to be misguided by remissions. Notwithstanding we are sure as to the final result, yet it is our duty to mitigate the patient's sad state as much as is in our power, and ever to seek to obtain those remissions which somewhat resemble the quiet, fertile spot in the arid desert. The mariner, tossing to and fro at the mercy of wind and waves, would gladly hail a calm though he were sure it was to be followed by a tempest that would certainly engulf him.

We must remember that however bad may appear the condition of our patient, yet at the eleventh hour has often come a change, making the victim, as it were, a new man — alas! to fall again. The possibility of the terrible symptoms disappearing to a great degree, and of this mad destroyer's lulling in its course for a time, should ever be sufficient to encourage the physician, were there nothing else to cheer him on.

As soon as possible, the patient should be removed to some quiet, comfortable, well regulated asylum, where he may be removed from the busy scenes of life, and the cares of his profession. We must counsel friends that the sooner this is done the more chance the patient has; and the longer it is delayed the more rapid will be his disease; and that left alone, he will commit, sooner or later, some scandalous or dangerous act at an unexpected moment.

When once the patient is confided to the physician, then he must ever have uppermost in his mind that, though he is not to effectually annihilate its progress and march, yet he now has to work; as has well remarked M. Calmeil, "Its degree of gravity depends on the extent of cerebral surface the disease occupies, whether in width, length, or depth."

Now must we bring into play all the resources of good hygiene, by furnishing sufficient alimentation, but not too rich in succulent, animal aliment. At an early hour must attention be given to not allowing the circulation to be so active as to produce congestion of the nervous centres. If the patient is robust and plethoric, we may employ local bleeding to a moderate degree by the application of leeches to the nasal fossæ, anus, neck or ears. The cupping glass may be used if more convenient. If we desire to recall an ancient hæmorrhoidal flux, or the suppression of the menses, the leech should be applied at the anus or vulva.

If the tendency to cerebral congestion is accompanied with agitation, petulance, or furor, it will be well to often employ baths, which may be prolonged three or four hours, according to necessity. The cold water bath by affusion, the sulphurous bath, and others which prompt reaction of the circulation towards the surface of the body, are preferable to quell the hyperæmic state. During and after the bath, application of cold compresses to the head is strongly recommended.

Continued attention must be given to the alimentary canal; constipation in this disease is constantly present, and also tends to increase congestion. To counteract this latter, the salts of soda and magnesia, and castor oil, are greatly in favor. Jalap alone, or with calomel, is much used.

The use of opium and other narcotics is questionable as to rendering service, and should, if used, be carefully employed. If the disease has assumed the form of melancholia or dementia, then we must be cautious in the employment of bleeding, possibly putting it completely aside; cathartics here, as in other forms, are often necessary. If the patient is troubled with torpor, but is not too much reduced, often the application of a moxa or a cautery is beneficial.

In some cases there is such a degree of anæmia, produced by melancholia, refusal of food, or various excesses, as to require the use of tonic treatment instead of depletion. Cod liver oil, iron, and quinine, then are useful. M. Marcé, at Bicetre — a hospital at Paris — has profitably used iodine and arsenic in small doses, when the digestive organs were inactive.

The activity of the antiphlogistic system should be lessened when the loss of memory, obliteration of the mental faculties, and difficulty of speech continue to increase, as we then see that the malady is attacking new regions in spite of all our endeavors to prevent it.

Now, as the second and third periods have arrived, the cerebral substance being disorganized, all we can do is to administer to the wants of the patient; sustain him as much as possible, by hygiene; give proper attention to the bowels, using all possible means to preserve his person and clothes in a clean condition, thus endeavoring to avoid, as long as possible, the formation of eschars; if necessary, use the catheter in case of urinary retention; compel the persons attending to give him such food, in so fine a state, that there shall be no accidents from imperfect mastication and difficult deglutition.

When congestive attacks assume an apoplectiform or epileptiform nature, and there is immediate danger, then must the usual means be employed to dissipate the momentary danger.

From the beginning to the fatal termination, let kindness rule in every word and every action.

